# Sistema de apoyo a las decisiones clínicas basado en el conocimiento para clasificar automáticamente la anemia de pacientes en hemodiálisis

**DOI:** 10.7705/biomedica.7945

**Published:** 2025-12-10

**Authors:** Sebastián Tenorio, Luis Alfonso Valderrama, José Javier Arango, Luz Amparo Lozano, Iván Leonardo Mojica

**Affiliations:** 1 SYNLAB Colombia, Bogotá, D. C., Colombia SYNLAB Colombia Bogotá D. C Colombia; 2 Clínica Imbanaco, Cali, Colombia Clínica Imbanaco Cali Colombia; 3 Asociación Colombiana de Medicina Interna - ACMI, Bogotá, D. C., Colombia Asociación Colombiana de Medicina Interna - ACMI Bogotá D. C Colombia

**Keywords:** anemia, insuficiencia renal crónica, diálisis renal, sistemas especialistas, sistemas de apoyo a las decisiones clínicas, ferritinas, hormona paratiroidea, Anemia, renal insufficiency, chronic, renal dialysis, expert systems, decision support systems, clinical, ferritins, parathyroid hormone

## Abstract

**Introducción.:**

La anemia es una complicación frecuente de los pacientes con enfermedad renal crónica y en hemodiálisis, asociada con mayor morbimortalidad y uso de recursos. Su clasificación adecuada es esencial para optimizar el tratamiento con hierro intravenoso y agentes estimulantes de la eritropoyesis. Los sistemas de apoyo a las decisiones clínicas basados en el conocimiento permiten estandarizar esta clasificación.

**Objetivo.:**

Describir el desarrollo y el funcionamiento de un sistema de apoyo a las decisiones clínicas -basado en el conocimiento- para la clasificación automatizada de la anemia de los pacientes en hemodiálisis, utilizando datos reales de laboratorio.

**Materiales y métodos.:**

Se llevó a cabo un estudio observacional retrospectivo de 883 pacientes adultos en hemodiálisis prevalente durante el 2023. Se construyó un algoritmo con base en las guías clínicas de la Sociedad Latinoamericana de Nefrologia e Hipertensión (SLANH), *Kidney Disease: Improving Global Outcomes* (KDIGO), *National Institute for Health and Care Excellence* (NICE) para clasificar a los pacientes con hemoglobina menor de 12 g/dl en tres categorías: déficit absoluto de hierro, déficit funcional de hierro y candidatos a prueba terapéutica con hierro intravenoso. También, se identificaron los casos con sospecha de hiperparatiroidismo secundario grave [paratohormona (PTH) mayor de 800 pg/ml]. Se usaron los datos del sistema de laboratorio y del sistema de apoyo a las decisiones clínicas, y se analizaron con estadísticas descriptivas.

**Resultados.:**

El sistema de apoyo a las decisiones clínicas clasificó a los pacientes en los siguientes grupos: déficit funcional de hierro (39,2 %), hiperparatiroidismo secundario grave (26,7 %), déficit absoluto de hierro (17,7 %) y candidatos a la prueba terapéutica (16,4 %). Un subgrupo (9,5 % con déficit funcional de hierro) presentó elevación de la PTH, lo cual sugiere resistencia a los agentes estimulantes de la eritropoyesis. Se observaron diferencias clínicas entre los grupos.

**Conclusiones.:**

El sistema de apoyo a las decisiones clínicas permitió hacer una clasificación automatizada de la anemia en hemodiálisis, apoyando aquellas basadas en la evidencia. Su implementación representa un avance en la salud digital, con potencial para mejorar la calidad del manejo de la enfermedad renal crónica.

## Abstract (en)

**Introduction.:**

Anemia is a frequent complication in patients with chronic kidney disease undergoing hemodialysis and is associated with increased morbidity, mortality, and healthcare burden. Accurate classification is essential to optimize treatment with intravenous iron and erythropoiesis-stimulating agents. Rule-based clinical decision support systems (CDSS) provide a strategy to standardize this process.

**Objective.:**

To describe the development and implementation of a knowledge-based clinical decision support system for the automated classification of anemia in hemodialysis patients using laboratory data.

**Materials and methods.:**

This retrospective observational study included 883 adult patients receiving prevalent hemodialysis during 2023. An algorithm was developed based on established clinical guidelines *[Sociedad Latinoamericana de Nefrología e Hipertensión* (SLANH)], KDIGO, NICE to classify patients with hemoglobin below 12 g/dl into three categories: absolute iron deficiency, functional iron deficiency, and candidates for therapeutic trial with intravenous iron. The system also flagged cases with suspected severe secondary hyperparathyroidism (PTH > 800 pg/ml). Data was obtained from the laboratory information system and the clinical decision support system. We applied a descriptive statistical analysis.

**Results.:**

The clinical decision support system automatically classified patients into the following categories: functional iron deficiency (39.2%), severe hyperparathyroidism (26.7%), absolute iron deficiency (17.7%), and candidates for intravenous iron trial (16.4%). A subgroup (9.5% within the functional iron deficiency group) also showed elevated PTH levels, suggesting potential resistance to erythropoiesis-stimulating agents. Distinct clinical profiles were observed across the groups.

**Conclusions.:**

The clinical decision support system enabled automated and standardized classification of anemia in hemodialysis patients, supporting evidence-based clinical decision-making. Its implementation represents a digital health innovation with the potential to improve the quality and safety of anemia management in chronic kidney disease.

La enfermedad renal crónica constituye un importante problema de salud pública a nivel global. Un análisis de la carga global de la enfermedad (GBD2021) estimó que en el 2021 había 673,7 millones de personas con esta enfermedad, lo que equivale, aproximadamente, al 8,54 % de la población mundial. Este estudio demostró un incremento del 92 % del número de casos desde 1990 y un aumento en la tasa de incidencia estandarizada por edad, que pasó de 192,45 a 233,65 por cada 100.000 habitantes en tres décadas; los decesos asociados a la enfermedad renal crónica alcanzaron 18,3 por 100.000 habitantes, con más de 41 millones de años de vida ajustados por discapacidad perdidos en el 2019 ^1^.

La enfermedad renal crónica ha escalado de forma sostenida en el *ranking* global de mortalidad, ya que pasó del puesto 19 en 1990 al 11 en el 2019 y se proyecta que será la quinta causa de muerte para el 2040 ^2^. En Latinoamérica, la prevalencia fue del 9,9 % en el 2021, mientras que en Colombia alcanzó el 10,7 % ^3^.

Entre las complicaciones más frecuentes y relevantes de la enfermedad renal crónica, se encuentra la anemia, cuya prevalencia en los pacientes en estadio 5 puede superar el 50 % ^4^. Esta condición contribuye significativamente a la disminución de la calidad de vida, el incremento del riesgo cardiovascular y la progresión a enfermedad renal terminal ^5^^,^^6^. La anemia en la enfermedad renal crónica tiene una fisiopatología multifactorial que incluye deficiencia de eritropoyetina, inflamación crónica, aumento de la hepcidina y alteraciones del metabolismo del hierro ^5^. Existe una correlación inversa bien documentada entre el descenso de la tasa de filtración glomerular y los niveles de hemoglobina ^6^^,^^7^.

Tradicionalmente, el manejo de la anemia se ha centrado en el uso de agentes estimulantes de la eritropoyesis y hierro intravenoso. Sin embargo, diversos estudios han documentado respuestas subóptimas al tratamiento, atribuibles principalmente al déficit funcional de hierro y a la presencia de inflamación, que pueden llevar a un uso inadecuado de los agentes estimulantes de la eritropoyesis con riesgos asociados como hipertensión y eventos trombóticos ^8^^,^^9^. Esta brecha entre las recomendaciones de las guías y la práctica clínica, plantea la necesidad de estrategias que favorezcan una toma de decisiones más estandarizada y segura.

En este contexto, el sistema de apoyo a las decisiones clínicas *(Clinical Decision Support System,* CDSS), desarrollado en este estudio, contribuye a cerrar dicha brecha al automatizar la aplicación de criterios diagnósticos basados en guías actualizadas y parámetros objetivos de laboratorio. De este modo, el sistema permite identificar de manera estandarizada los diferentes perfiles de anemia (incluidos el déficit funcional de hierro y la coexistencia de inflamación), y generar recomendaciones individualizadas para el uso racional del hierro intravenoso y de los agentes estimulantes de la eritropoyesis. Así, este brinda apoyo al equipo clínico en la toma de decisiones seguras y alineadas con la evidencia para reducir la variabilidad en la práctica y minimizar los riesgos asociados al manejo inadecuado.

En este caso, los sistemas de apoyo a las decisiones clínicas representan herramientas digitales emergentes que pueden contribuir a mejorar la observancia de las guías, reducir la variabilidad en la práctica clínica y optimizar el tratamiento de la anemia en el contexto de la enfermedad renal crónica. Estos sistemas pueden clasificarse como basados en el conocimiento -que utilizan reglas de tipo "si-entonces"- o impulsados por datos, como los que emplean el aprendizaje automático ^10^.

Este estudio presenta el desarrollo de un algoritmo dentro de un sistema de apoyo a las decisiones clínicas basado en el conocimiento, para asistir en el diagnóstico y el manejo de la anemia de los pacientes en hemodiálisis. Las recomendaciones fueron construidas a partir de aquellas de la Sociedad Latinoamericana de Nefrología e Hipertensión (SLANH, versión 2018) y las de otras guías internacionales (KDIGO 2012, *European Renal Best Practice* - ERBP 2013, NICE 2015), y sus actualizaciones recientes. El algoritmo clasifica pacientes con hemoglobina menor de 12 g/dl en función del perfil férrico: identifica casos de deficiencia absoluta de hierro (ferritina < 200 ng/ ml y TSAT < 20 %), candidatos a prueba terapéutica con hierro intravenoso (ferritina 200-500 ng/ml y TSAT 20-30 %) e incorpora alertas por hormona paratiroidea (PTH) elevada.

En el diseño del algoritmo, se priorizó la guía de la Sociedad Latinoamericana de Nefrología e Hipertensión (SLANH, 2018), que refleja las realidades y necesidades clínicas de la región, e incluye variables de acceso y epidemiología locales. Las guías internacionales (KDIGO, ERBP, NICE) se emplearon de manera complementaria para fortalecer y actualizar los criterios diagnósticos y de manejo no abordados específicamente por la SLANH, o para incorporar recomendaciones con evidencia suficiente pero que aún no han sido adaptadas a nivel regional. Así, el sistema de apoyo a las decisiones clínicas integra lo mejor de la evidencia internacional, aunque prioriza las recomendaciones de la guía regional, lo cual garantiza la pertinencia local y la aplicabilidad clínica en el contexto colombiano y latinoamericano.

Este estudio tuvo como objetivo evaluar la implementación de un sistema de apoyo a las decisiones clínicas mediante un análisis retrospectivo observacional de los datos de laboratorio clínico recolectados en el 2023. Se espera que este enfoque contribuya a la transformación digital de la práctica nefrológica, promoviendo decisiones clínicas basadas en la evidencia y orientadas a mejorar los desenlaces en salud de una población muy vulnerable.

## Materiales y métodos

### Diseño del estudio

Se llevó a cabo un estudio observacional y retrospectivo, cuyo objetivo fue describir el desarrollo e implementación de un sistema de apoyo a las decisiones basado en el conocimiento *(Clinical Decision Support Systems,* CDSS), aplicado al diagnóstico y subsecuente clasificación de la anemia de los pacientes adultos con enfermedad renal crónica en tratamiento con hemodiálisis prevalente (por más de 90 días continuos en el programa). El análisis se basó en los datos de laboratorio recolectados entre enero y diciembre del 2023.

Dado su diseño, no se planteó la validación cuantitativa mediante sensibilidad, especificidad u otros parámetros para medir el desempeño, ya que el propósito fue describir la implementación del motor de reglas clínicas, no evaluar su precisión diagnóstica.

La clasificación automatizada se basó en los siguientes criterios: los pacientes con ferritina sérica inferior a 200 ng/ml y saturación de transferrina menor del 20 %, fueron clasificados en el grupo con déficit absoluto de hierro; aquellos con ferritina entre 200 y 500 ng/ml y saturación de transferrina menor del 20 %, fueron incluidos en el grupo con déficit funcional de hierro; los pacientes con ferritina entre 200 y 500 ng/ml y saturación de transferrina entre el 20 y el 30 %, fueron identificados como candidatos a la prueba terapéutica con hierro intravenoso, y aquellos con niveles de hormona paratiroidea (PTH) superiores a 800 pg/ml, fueron clasificados con sospecha de hiperparatiroidismo secundario grave. Cada una de estas categorías generaba una recomendación clínica automatizada orientada a guiar el tratamiento o la reevaluación del paciente, estandarizando el proceso de estratificación basado en parámetros objetivos de laboratorio.

### Contexto del estudio y del equipo investigador

El estudio se desarrolló en el contexto del sistema de salud colombiano; incluyó datos de los pacientes atendidos en las unidades renales ambulatorias para la hemodiálisis crónica en distintas regiones del país. La cohorte analizada corresponde a personas mayores de edad con enfermedad renal crónica en estadio 5, en tratamiento sustitutivo mediante hemodiálisis, bajo esquemas de atención integral regidos por normas nacionales.

El equipo multidisciplinario responsable del desarrollo, revisión e implementación del sistema de apoyo a las decisiones clínicas, estuvo compuesto por profesionales de medicina interna, nefrología, patología clínica, salud pública y epidemiología clínica, quienes participaron en el diseño de las recomendaciones clínicas, la validación del algoritmo y la interpretación de los hallazgos.

Dado el carácter retrospectivo del análisis y el uso exclusivo de datos anonimizados provenientes del Sistema de Información de Laboratorio, no se requirió obtención de consentimiento informado, de acuerdo con los directrices éticas establecidas en la Resolución 8430 de 1993 del Ministerio de Salud de Colombia.

### Población y muestra

Los datos provienen de pacientes atendidos en las unidades de hemodiálisis de Colombia durante el 2023. La cohorte inicial incluyó 4.721 adultos con, al menos, un registro de laboratorio rutinario disponible.

Se aplicaron los siguientes criterios de exclusión: pacientes menores de 18 años, con hemoglobina mayor de 12 g/dl o con registros incompletos en las variables clave para la clasificación automatizada (hemoglobina, ferritina y saturación de transferrina). La identificación y la depuración de la muestra se llevaron a cabo mediante un filtro automatizado en el Sistema de Información de Laboratorio enlazado con el sistema de apoyo a las decisiones clínicas.

Tras aplicar estos criterios, la muestra final quedó conformada por 883 pacientes, todos en tratamiento de hemodiálisis prevalente. Estos pacientes fueron clasificados automáticamente por el algoritmo del sistema de apoyo a las decisiones clínicas según los criterios diagnósticos establecidos. La distribución por grupos clínicos fue: 346 con déficit funcional de hierro, 156 con déficit absoluto de hierro, 145 candidatos a la prueba terapéutica con hierro intravenoso y 236 pacientes con sospecha de hiperparatiroidismo secundario grave.

### Unidad de análisis

Cada paciente se consideró una unidad de análisis. Para cada sujeto se consideró el primer conjunto de resultados registrado en el 2023, cumpliendo con los criterios operacionales del algoritmo. Se garantizó que cada observación fuera única, sin duplicados ni múltiples episodios por paciente.

### Definición operacional de variables

Las variables incluidas fueron:

- Edad: años cumplidos al momento del registro,
- Sexo: femenino o masculino,
- Hemoglobina (Hb): g/dl (se definió anemia como Hb menor de 12 g/dl),
- Ferritina: ng/ml,
- Saturación de transferrina: porcentaje y
- Hormona paratiroidea (PTH): pg/ml.

Las clasificaciones clínicas derivadas del sistema de apoyo a las decisiones clínicas fueron:

- Déficit absoluto de hierro: ferritina menor de 200 ng/ml y saturación de transferrina menor de 20 %,
- Déficit funcional de hierro: ferritina entre 200 y 500 ng/ml y saturación de transferrina menor de 20 %,
- Candidato a la prueba terapéutica con hierro intravenoso: ferritina entre 200 y 500 ng/ml, y saturación de transferrina entre el 20 y el 30 %, y
- Sospecha de hiperparatiroidismo secundario grave: PTH mayor de 800 pg/ml.

### Definición del algoritmo del sistema de apoyo a las decisiones clínicas

El sistema de apoyo a las decisiones clínicas fue diseñado como un sistema experto basado en el conocimiento, con recomendaciones de tipo "si-entonces" derivadas de la guía de anemia de la Sociedad Latinoamericana de Nefrología e Hipertensión (SLANH, versión 2018), así como de las guías internacionales KDIGO 2012, ERBP 2013, NICE 2015 y actualizaciones recientes.

Las indicaciones o las recomendaciones se establecieron mediante consenso clínico con expertos en nefrología y fueron validadas antes de su implementación. El sistema clasifica automáticamente a los pacientes con hemoglobina menor de 12 g/dl en función del perfil férrico y emite recomendaciones clínicas. Además, genera una alerta para la sospecha de hiperparatiroidismo secundario grave, en caso de detectar niveles de PTH por encima de 800 pg/ml.

A diferencia de la interpretación manual de la guía clínica, el sistema de apoyo a las decisiones clínicas traduce los algoritmos diagnósticos y terapéuticos en indicaciones o recomendaciones lógicas, estructuradas y automatizadas, lo cual garantiza su aplicación uniforme a todos los pacientes, sin depender de la interpretación subjetiva o de la experiencia del profesional. Esta sistematización minimiza los errores humanos, las omisiones y las variabilidades en la aplicación de los criterios, lo cual garantiza que cada paciente sea evaluado bajo los mismos estándares objetivos establecidos por la evidencia y el consenso de expertos. Además, dicho sistema permite procesar grandes volúmenes de información en tiempo real, identificar patrones complejos y generar alertas automáticas que podrían pasar desapercibidas en la práctica clínica habitual, lo cual potencia la seguridad y la calidad en la toma de decisiones.

El sistema de apoyo a las decisiones clínicas es una herramienta tecnológica que permite asistir al profesional de la salud en la toma de decisiones clínicas, por medio de la integración de las fuentes de información, la aplicación de recomendaciones basadas en la evidencia y la generación de indicaciones específicas para cada paciente.

Diversos estudios han documentado que la simple existencia de guías clínicas no garantiza su adecuada implementación en la práctica, debido a factores como la sobrecarga asistencial, la variabilidad en la experiencia del personal y la complejidad de los algoritmos recomendados (10). Esto puede llevar a omisiones, interpretaciones incorrectas o retrasos en la aplicación de las recomendaciones, lo que afecta negativamente la calidad y la seguridad de la atención. El sistema de apoyo a las decisiones clínicas contribuye a mejorar esta situación, al generar recomendaciones automáticamente con las guías, lo cual garantiza que todos los pacientes sean evaluados de manera sistemática y uniforme, facilita la observancia de las recomendaciones, y reduce la brecha entre la evidencia y la práctica clínica.

En el caso particular del estudio descrito, el sistema de apoyo a las decisiones clínicas se nutre exclusivamente del *Sistema de información de laboratorio,* a partir del cual se extraen datos estructurados relacionados con los resultados de pruebas de laboratorio. Esta información se utiliza para activar reglas automatizadas de análisis clínico.

El sistema de apoyo a la decisión clínica, AlinIQCDS (Abbott, tecnología Beamtree RippleDown), utiliza un motor de reglas de tipo *rippledown rules,* diseñado para facilitar la actualización progresiva del conocimiento experto y la interpretación automatizada de los resultados. En este estudio, la integración con el LIS WinsisLab® se realizó mediante estándares internacionales como HL7, versión 2 y 3, y mapeo semántico de pruebas *(Logical Observation Identifiers Names and Codes,* LOINC) y códigos internos, lo que garantiza interoperabilidad estructural y compatibilidad semántica con otros sistemas de información de laboratorio. Gracias a esta arquitectura, el sistema de apoyo a las decisiones clínicas puede recibir los datos de laboratorio en tiempo real, evaluarlos automáticamente bajo las indicaciones clínicas establecidas y emitir recomendaciones o alertas personalizadas. Esto facilita su escalabilidad y replicabilidad en diferentes entornos clínicos.

Estas recomendaciones fueron previamente establecidas y revisadas por expertos, y se diseñaron con base en las guías de práctica clínica. Su automatización permite identificar situaciones clínicas relevantes, como valores fuera de rango, alteraciones en los valores de laboratorio o necesidad de practicar pruebas complementarias. Una vez activada su aplicación, el sistema de apoyo a las decisiones clínicas genera alertas personalizadas. En este estudio, dichas alertas se enviaron directamente por correo electrónico al grupo de expertos clínicos (directores médicos de las unidades renales), quienes revisaron los reportes generados y su interpretación. Esta validación permite emitir recomendaciones específicas para el manejo de cada caso.

### Descripción formal de las recomendaciones implementadas por el sistema de apoyo a las decisiones clínicas

El motor de indicaciones del sistema de apoyo a las decisiones clínicas, fue construido a partir de las guías vigentes de práctica clínica (SLANH 2018, KDIGO 2012, NICE 2015) y fue ajustado mediante el consenso de expertos. Las recomendaciones fueron expresadas mediante lógica condicional estructurada, evaluando los parámetros de laboratorio de pacientes adultos con valores de hemoglobina menores de 12 g/dl.

A continuación, se describen formalmente las recomendaciones implementadas:

*• Anemia por déficit absoluto de hierro:*

Pacientes con 18 o más años de edad, hemoglobina menor de 12 g/dl, saturación de transferrina menor del 20 % y ferritina sérica menor de 200 ng/ml.

Algoritmo lógico:

(Edad ≥ 18) AND (Hemoglobina < 12) AND (TSAT < 20) AND (Ferritina < 200)

*• Anemia por déficit funcional de hierro:*

Pacientes con 18 o más años, hemoglobina menor de 12 g/dl, saturación de transferrina menor de 20 % y ferritina entre 200 y 500 ng/ml.

Algoritmo lógico:

(Edad ≥ 18) AND (Hemoglobina < 12) AND (TSAT < 20) AND (Ferritina ≥ 200 AND Ferritina ≤ 500)

*• Candidatos a prueba terapéutica con hierro intravenoso:*

Pacientes con 18 o más años, hemoglobina menor de 12 g/dl, saturación de transferrina entre el 20 y el 30 %, y ferritina entre 200 y 500 ng/ml.

Algoritmo lógico:

(Edad ≥ 18) AND (Hemoglobina < 12) AND (TSAT ≥ 20 AND TSAT ≤ 30) AND (Ferritina ≥ 200 AND Ferritina ≤ 500)

Estas recomendaciones fueron codificadas e implementadas dentro del sistema de apoyo a las decisiones clínicas que, después fue aplicado de forma automática a registros únicos de laboratorio clínico, permitiendo la clasificación y la emisión de alertas clínicas específicas.

### Recolección y gestión de los datos

Los datos fueron extraídos de dos fuentes principales: ^1^ el sistema de información de laboratorio llamado WinsisLab, versión 7, que proporcionó los resultados de hemoglobina, ferritina, saturación de transferrina y PTH; y ^2^ el sistema de apoyo a las decisiones clínicas llamado AlinIQCDS, versión 8.2.10, que generó automáticamente las recomendaciones. Posteriormente, ambas fuentes fueron consolidadas en una única base de datos, previa verificación de correspondencia entre el paciente y la fecha de recolección. Se llevó a cabo un control de calidad mediante la revisión manual del 10 % de los registros seleccionados aleatoriamente, con validación extendida en caso de discrepancias.

### Manejo de datos faltantes

Se utilizó un enfoque de análisis por casos completos. Aquellos pacientes con registros faltantes en alguna de las variables esenciales para la clasificación (hemoglobina, ferritina o saturación de transferrina), fueron excluidos del análisis. No se aplicaron métodos de imputación dado que el sistema de apoyo a las decisiones clínicas requiere información completa para generar sus recomendaciones.

### Análisis estadístico

Se hizo un análisis descriptivo. Las variables cuantitativas se resumieron mediante la media y su desviación estándar, la mediana y rangos intercuartílicos. Las variables categóricas se expresaron como frecuencias absolutas y proporciones. No se hicieron análisis inferenciales dado que el objetivo del estudio fue caracterizar la clasificación generada por el sistema de apoyo a las decisiones clínicas sin establecer asociaciones causales. El procesamiento y visualización se llevaron a cabo utilizando Microsoft Excel® y Python, versión 3.11, con las librerías pandas, numpy y matplotlib.

### Consideraciones éticas

Este estudio fue clasificado como una investigación sin riesgo, de acuerdo con la Resolución 8430 de 1993 del Ministerio de Salud de Colombia. Se utilizaron datos previamente recolectados y anonimizados. Se implementaron medidas estrictas para proteger la confidencialidad de los participantes. Todos los investigadores del estudio cuentan con formación científica y ética acorde con los principios de la Declaración de Helsinki.

## Resultados

Durante el 2023, 883 pacientes adultos en tratamiento con hemodiálisis por más de 90 días seguidos, hemodiálisis prevalente fueron evaluados mediante el sistema de apoyo a las decisiones clínicas diseñado para el diagnóstico y la clasificación de la anemia. La edad media de la cohorte fue de 54,5 años (desviación estándar, DE ± 16,3), con una proporción de mujeres del 43,5 %.

- Con base en los criterios operacionales definidos por el algoritmo, los pacientes fueron clasificados automáticamente en los siguientes grupos clínicos:
- *Déficit funcional de hierro:* 346 pacientes (39,2 %), edad media de 58,5 años (DE ± 16,4), el 43,6 % eran mujeres; hemoglobina media = 10.4 g/dl; ferritina = 369,2 ng/ml; saturación de transferrina = 14,1 %.
- *Déficit absoluto de hierro:* 156 pacientes (17,7 %), edad media de
- 57.5 años (DE ± 15,3), el 39,1 % eran mujeres; hemoglobina media = 10,2 g/dl; ferritina = 118,7 ng/ml; saturación de transferrina = 11,3 %.
- *Candidatos a la prueba terapéutica con hierro intravenoso:* 145 pacientes (16,4 %), edad media de 59,2 años (DE ± 16,8), el 34,5 % eran mujeres; hemoglobina media = 10,7 g/dl; ferritina = 372,5 ng/ml; saturación de transferrina = 23,6 %.
- *Sospecha de hiperparatiroidismo secundario grave:* 236 pacientes (26,7 %), edad media de 47,5 años (DE ± 16,0), el 50,0 % eran mujeres; PTH media = 1.378,5 pg/ml.

**Figura 1:**
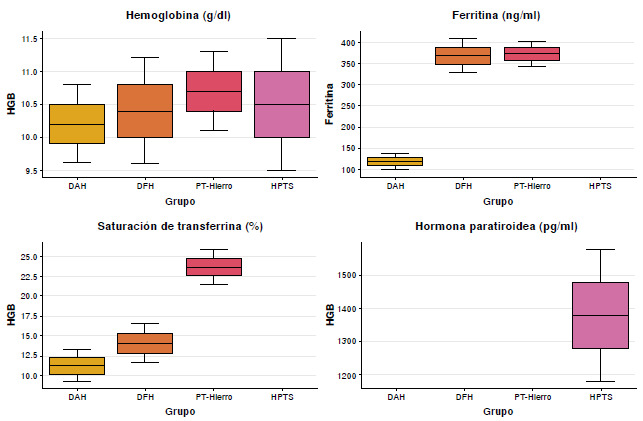
Distribución de las variables clínicas por grupo de clasificación del *sistema de apoyo a las decisiones clínicas* en los pacientes con anemia durante la hemodiálisis

Las características clínicas por grupo se resumen en el cuadro 1. La clasificación automatizada del sistema de apoyo a las decisiones clínicas permitió segmentar la población en subgrupos con perfiles fisiopatológicos diferenciables, como se observa en la figura 1, donde se representa la distribución de las variables continuas clave (hemoglobina, ferritina, saturación de transferrina, PTH) mediante gráficos de caja.

**Cuadro 1:** Características clínicas de los pacientes clasificados por el sistema de apoyo a las decisiones clínicas para el diagnóstico y el manejo de la anemia en hemodiálisis

Grupo clínico	n	Edad media (DE)	Mujeres (%)	Hb media (g/dl)	Ferritina media (ng/ml)	Saturación media de transferrina (%)	PTH media (pg/ml)
Déficit absoluto de hierro	346	58,5 (16,4)	43,6	10,4	369,2	14,1	-
Déficit funcional de hierro	156	57,5 (15,3)	39,1	10,2	118,7	11,3	-
Candidatos a prueba terapéutica con hierro intravenoso	145	59,2 (16,8)	34,5	10,7	372,5	23,6	-
Sospecha de hiperparatiroidismo secundario grave	236	47,5 (16,0)	50,0	-	-	-	1378,5

DE: desviación estándar; Hb: hemoglobina; TSAT: saturación de transferrina; PTH: hormona paratiroidea

Los valores que no estaban disponibles se indican con guiones (-).

En la figura 1, se aprecia que los pacientes con déficit absoluto de hierro presentaron las concentraciones más bajas de ferritina y saturación de transferrina, en contraste con los candidatos a la prueba terapéutica con hierro intravenoso, quienes tenían valores intermedios de ferritina, pero con una saturación elevada de transferrina. Por su parte, el grupo con déficit funcional de hierro presentó ferritina elevada con baja saturación de transferrina, patrón característico de la inflamación crónica. El grupo con sospecha de hiperparatiroidismo secundario grave presentó concentraciones de hemoglobina comparables a la de los otros subgrupos, pero con niveles elevados de hormona paratiroidea y una mayor dispersión de los valores.

Además, se identificó un subconjunto de 33 pacientes (9,5 % del grupo con déficit funcional de hierro) que también presentaba niveles elevados de PTH (mayor de 800 pg/ml). Esta coocurrencia podría indicar una interacción clínica relevante entre inflamación, metabolismo mineral alterado y resistencia a la eritropoyetina, lo cual refuerza la utilidad del sistema de apoyo a las decisiones clínicas para detectar combinaciones de riesgo clínico.

En este sentido, dicho sistema no se limita únicamente a replicar las recomendaciones propuestas en las guías clínicas, sino que, gracias a su capacidad para analizar múltiples parámetros de laboratorio de forma simultánea, puede identificar combinaciones atípicas o concurrencias de alteraciones bioquímicas (como el déficit funcional de hierro junto con hiperparatiroidismo grave), que podrían pasar desapercibidas en la práctica rutinaria. Aunque las recomendaciones se derivan de las guías clínicas, la automatización y el análisis sistemático que ofrece este sistema de apoyo permite detectar patrones relevantes de riesgo clínico y generar alertas integradas que apoyan la toma individualizada y proactiva de decisiones, lo cual facilita intervenciones más oportunas en situaciones complejas.

Se presentan gráficos de caja para los valores de hemoglobina, ferritina sérica, saturación de transferrina y hormona paratiroidea, estratificados según la clasificación automatizada del sistema de apoyo a las decisiones clínicas: déficit absoluto de hierro, déficit funcional de hierro, candidatos a la prueba terapéutica con hierro intravenoso y sospecha de hiperparatiroidismo secundario grave. En la figura 1 se muestran las diferencias en los perfiles hematológicos y hormonales entre los subgrupos clínicos, lo que refuerza la estratificación individualizada del tratamiento.

El presente estudio tuvo como objetivo el desarrollo, la sistematización y la automatización de la clasificación de la anemia en los pacientes en hemodiálisis prevalente, mediante un sistema de apoyo a las decisiones clínicas basado en recomendaciones explícitas derivadas de guías de práctica clínica (SLANH, KDIGO, NICE).

Dado el diseño observacional y descriptivo del estudio, no se planteó la validación cuantitativa de la clasificación automatizada mediante indicadores de medición de parámetros como sensibilidad, especificidad, valor predictivo positivo o valor predictivo negativo. Tampoco, se hicieron análisis de concordancia con evaluadores clínicos, ya que el propósito no fue establecer un nuevo método diagnóstico, sino describir la implementación y la factibilidad de un sistema de apoyo a las decisiones médicas basado en el conocimiento clínico estandarizado.

Para mayor claridad, el sistema implementado está compuesto de tres módulos principales: 1) el LIS WinsisLab®, que recopila y almacena los resultados de laboratorio de cada paciente en un formato estructurado; 2) el sistema de apoyo a las decisiones clínicas AlinIQCDS (Abbott Diagnostics, tecnología *rippledown),* que recibe automáticamente estos datos a través de mensajes HL7 y mapeo de identificadores de pruebas, y 3) el módulo de notificación, encargado de enviar por correo electrónico seguro las alertas y las recomendaciones generadas por el sistema de apoyo a las decisiones clínicas al equipo clínico. El flujo operativo consiste en la extracción programada de los datos del *Sistema de información de laboratorio,* su procesamiento en tiempo real por el motor de reglas del sistema de apoyo a las decisiones clínicas, la generación de las recomendaciones clínicas individualizadas y el envío automatizado de reportes al equipo médico. Esta arquitectura modular, basada en estándares de interoperabilidad (HL7, LOINC), permite escalar y adaptar el modelo a otros entornos con sistemas compatibles de información de laboratorio.

Las futuras investigaciones deberán evaluar el impacto clínico del modelo, la precisión de las clasificaciones en las condiciones de la práctica clínica real y la implementación del sistema por parte del equipo tratante.

## Discusión

En este estudio se describe el desarrollo y la implementación de un sistema de apoyo a las decisiones clínicas basado en el conocimiento, para la clasificación automatizada de la anemia en los pacientes con enfermedad renal crónica en hemodiálisis, el cual integra parámetros clave de laboratorio y guías clínicas vigentes. La aplicación del algoritmo permitió una estratificación automática de los pacientes en perfiles clínicos diferenciados, lo que facilita la toma de decisiones terapéuticas personalizadas en entornos de gran complejidad.

La anemia en la enfermedad renal crónica es una complicación frecuente, multifactorial y clínicamente significativa. Aunque típicamente es normocítica y normocrómica, debe considerarse como un diagnóstico de exclusión, ya que puede coexistir con otras causas hematológicas ^4^^,^^11^. En este contexto, la deficiencia de hierro, tanto absoluta como funcional, es una de las principales causas modificables y su manejo adecuado es esencial para optimizar la respuesta al tratamiento con agentes estimulantes de la eritropoyesis ^12^. Según la guía KDIGO 2024, los niveles de hemoglobina descienden de forma progresiva con la disminución de la tasa de filtración glomerular, especialmente en pacientes mayores de 65 años con tasa de filtración glomerular menor de 15 ml/min/1,73 m^2^, lo cual concuerda con los valores observados en nuestra cohorte ^11^. Desde su aprobación en 1989, la eritropoyetina humana recombinante transformó el tratamiento de la anemia en casos de enfermedad renal crónica ^13^^,^^14^.

El sistema de apoyo a las decisiones clínicas permitió establecer que el 39,2 % de los pacientes presentaba déficit funcional de hierro, seguido de sospecha de hiperparatiroidismo secundario grave (26,7 %) y déficit absoluto de hierro (17,7 %); el 16,4 % resultó candidato a la prueba terapéutica con hierro intravenoso. Estas proporciones reflejan la gran carga de inflamación crónica y alteraciones del metabolismo mineral en esta población. La identificación de 33 pacientes (9,5 % del grupo con déficit funcional de hierro) que presentaban niveles elevados de PTH (mayor de 800 pg/ml) representa un subgrupo de alto riesgo clínico, dado que el hiperparatiroidismo favorece la resistencia a los agentes estimulantes de la eritropoyesis, una causa frecuentemente subestimada en la práctica clínica ^15^.

Además del impacto clínico del sistema de apoyo a las decisiones clínicas en la estandarización de la clasificación de la anemia, es importante considerar los hallazgos recientes sobre las estrategias terapéuticas en hemodiálisis. Petzer *et al.* demostraron que el uso de un anticuerpo monoclonal anti-BMP6 (KY1070) puede mejorar la biodisponibilidad del hierro y reducir la necesidad de la eritropoyetina, una alternativa terapéutica prometedora en situaciones de poca respuesta a los agentes estimulantes de la eritropoyesis ^16^. Por otra parte, Kim *et al.* i observaron que la vía de administración subcutánea de la eritropoyetina, en comparación con la vía intravenosa, se asocia con mayor variabilidad de los niveles de hemoglobina y un riesgo incrementado de eventos cardiovasculares, lo que resalta la importancia de una adecuada selección de la vía de administración ^17^.

La literatura sugiere que la saturación de transferrina y la ferritina sérica, presentan limitaciones como biomarcadores únicos del estado férrico durante los procesos inflamatorios. La ferritina es una proteína de fase aguda cuyo valor puede elevarse sin reflejar reservas reales de hierro, lo cual dificulta la diferenciación entre los pacientes con déficit absoluto y aquellos con déficit funcional de hierro ^18^^,^^19^. En este sentido, el uso de un sistema de apoyo a las decisiones clínicas basado en indicaciones clínicas explícitas permite estandarizar los criterios y minimizar la subjetividad del juicio clínico en condiciones de gran complejidad.

En la práctica clínica, los valores elevados de ferritina pueden llevar a una interpretación errónea de las reservas adecuadas de hierro, ya que pueden estar enmascarando un déficit funcional, especialmente en los procesos de inflamación. El sistema de apoyo a las decisiones clínicas contribuye a resolver esta ambigüedad, al no basar sus recomendaciones en un único parámetro, sino en la evaluación conjunta de múltiples indicadores, como la saturación de transferrina, la ferritina, la hemoglobina y, cuando corresponde, la proteína C reactiva. Las recomendaciones clínicas implementadas están diseñadas para reconocer patrones compatibles con un déficit funcional de hierro, incluso si la ferritina está aparentemente normal o elevada y la saturación de transferrina está reducida. Esto reduce la interpretación subjetiva por parte del profesional y mejora la precisión diagnóstica en situaciones complejas.

El algoritmo implementado en este estudio se construyó a partir de las recomendaciones de la SLANH (2018), que definen el déficit absoluto de hierro como ferritina menor de 200 ng/ml y saturación de transferrina menor del 20 %, y el déficit funcional de hierro, como ferritina entre 200 y 500 ng/ml con saturación de transferrina menor del 20 %. Para los pacientes candidatos a la prueba terapéutica con hierro intravenoso, se utiliza un rango de saturación de transferrina entre el 20 y el 30 %, y de ferritina entre 200 y 500 ng/ml ^8^. Estos puntos de corte han sido validados en ensayos como el estudio PIVOTAL, en el cual la administración proactiva de hierro intravenoso en dosis altas redujo la necesidad de usar estimulantes de la eritropoyesis, las transfusiones y los eventos adversos, sin aumentar el riesgo cardiovascular ^20^.

Mark *et al.,* en un análisis preespecificado del estudio PIVOTAL, evaluaron el riesgo de accidentes cerebrovasculares asociados con diferentes estrategias de administración del hierro intravenoso, y concluyeron que una estrategia proactiva no incrementa dicho riesgo y podría ser segura en los pacientes en hemodiálisis ^21^.

De manera complementaria, Macdougall *et al.* reportaron que la administración de hierro intravenoso y de agentes de larga acción estimulantes de la eritropoyesis en pacientes en hemodiálisis frecuente, reduce la dosis total acumulada de estos agentes, sin comprometer el control de la anemia ^22^.

En línea con estos hallazgos, Petrie *et al.* reportaron que el tratamiento proactivo con hierro en dosis elevadas reduce significativamente la necesidad de administrar agentes estimulantes de la eritropoyesis y se asocia con menores riesgos de infarto agudo de miocardio, sin incrementar los eventos adversos mayores ^23^.

Finalmente, Singh *et al.* evaluaron el efecto de la sacarosa férrica sobre la presión arterial intradialítica. Los autores observaron que dosis de 100 mg o más se asociaban con una menor incidencia de hipotensión, aunque con un incremento de episodios de hipertensión durante la diálisis, lo que evidencia sus efectos hemodinámicos y la necesidad de monitoreo continuo ^24^.

Los resultados de estos estudios refuerzan la necesidad de contar con sistemas que permitan ajustar de forma individualizada las estrategias del manejo de la anemia. En este sentido, el sistema desarrollado de apoyo a las decisiones clínicas en este estudio representa una herramienta potencialmente útil para integrar los factores mencionados -como el esquema de la administración de hierro intravenoso, la dosificación de los agentes estimulantes de la eritropoyesis, los parámetros clínicos y de laboratorio relacionados con la respuesta al tratamiento y los riesgos hemodinámicos-a la práctica clínica diaria. En este sistema, la individualización se logra mediante la aplicación de parámetros condicionales que combinan múltiples variables clínicas y de laboratorio específicas por paciente (hemoglobina, ferritina, saturación de transferrina y PTH), evaluadas en tiempo real.

El sistema de apoyo a las decisiones clínicas utiliza un motor de reglas de tipo *ripple-down rules* que permite construir excepciones clínicas de forma jerárquica, mostrando situaciones particulares que requieren abordajes diferenciados. Por ejemplo, un paciente con hemoglobina de 10,5 g/dl, ferritina de 450 ng/ml y saturación de transferrina del 18 %, activa una regla compatible con el déficit funcional de hierro, lo que sugiere un cuadro inflamatorio con secuestro férrico.

Estos parámetros generan una alerta para reevaluar el estado inflamatorio, la necesidad y el tipo de suplementación férrica, y el ajuste de los agentes estimulantes de la eritropoyesis. Por otro lado, un paciente con hemoglobina de 11,8 g/dl, ferritina de 120 ng/ml y saturación de transferrina del 14 %, activa una regla compatible con déficit absoluto de hierro, para el cual se sugiere la reposición directa con hierro intravenoso. Asimismo, si un tercer paciente presenta hemoglobina baja y PTH mayor de 800 pg/ml, el sistema genera una alerta combinada que señala el riesgo de hiperparatiroidismo secundario grave, potencialmente asociado a la resistencia a la eritropoyetina. Así, el sistema de apoyo a las decisiones clínicas no emite recomendaciones genéricas, sino alertas adaptadas al perfil clínico y bioquímico individual, lo que promueve decisiones terapéuticas más precisas y seguras.

Persisten desafíos asociados con la poca respuesta al tratamiento. Las guías KDIGO definen la poca respuesta inicial y subsecuente según los cambios esperados de los niveles de hemoglobina una vez se administre la dosis estándar de agentes estimulantes de la eritropoyesis. Entre las causas, se destacan el déficit de hierro, la inflamación, las infecciones, el hiperparatiroidismo secundario y la malnutrición ^15^. En este sentido, la incorporación de un sistema de apoyo a las decisiones clínicas permite identificar el tipo de déficit y, también, alertar sobre condiciones comórbidas como el hiperparatiroidismo secundario grave, que podrían condicionar un manejo más intensivo y multidisciplinario.

Además, estudios como el de Barbieri *et al.* han demostrado que los sistemas de apoyo a las decisiones clínicas pueden mejorar significativamente la estabilidad de la hemoglobina, aumentar la proporción de pacientes dentro del rango objetivo y reducir la dosis media de los agentes estimulantes de la eritropoyesis, cuando sus recomendaciones son implementadas de forma sistemática ^25^. La posibilidad de integrar estas herramientas en los registros médicos electrónicos, permite una mayor observancia de las guías de práctica clínica, la reducción del error médico y la personalización de la atención, alineado en concordancia con los principios propuestos por la Organización Mundial de la Salud (OMS) para la salud digital ^1^^,^^10^.

Entre las fortalezas de este estudio, se encuentran la construcción de un algoritmo basado en la evidencia, la integración automatizada con el sistema de laboratorio, el análisis descriptivo por grupos clínicos diferenciados y la visualización gráfica de los resultados, lo cual facilita la transferencia de conocimiento a la práctica clínica.

Entre las principales limitaciones, se destaca su diseño observacional retrospectivo, lo que puede restringir la generalización de los resultados a otros entornos asistenciales. Asimismo, el análisis se fundamentó exclusivamente en variables de laboratorio, sin incorporar biomarcadores inflamatorios específicos, como la proteína C reactiva o la interleucina 6, que podrían haber enriquecido la evaluación del estado férrico en los contextos de inflamación crónica. Tampoco, se evaluaron desenlaces clínicos como la respuesta terapéutica, los eventos cardiovasculares o la supervivencia, ni se midió el cumplimiento del equipo médico a las recomendaciones emitidas por el sistema de apoyo a las decisiones clínicas.

En este sentido, si bien el estudio se enfocó en la construcción e implementación de motores de reglas clínicas aplicadas a datos retrospectivos extraídos del sistema de información de laboratorio, con el acompañamiento metodológico de expertos en nefrología y patología clínica, su alcance se restringió a la revisión de los resultados analíticos sin la integración de información clínica adicional. No obstante, esta experiencia representa un avance crucial hacia futuras fases de validación clínica prospectiva. Estas fases contemplan la incorporación de variables clínicas complementarias -como los antecedentes patológicos, las comorbilidades y los tratamientos farmacológicos activos- para enriquecer la interpretación contextualizada de los hallazgos, mejorar la precisión de las alertas generadas y ampliar la aplicabilidad del sistema de apoyo en situaciones reales de atención médica.

La implementación de un sistema de apoyo a las decisiones clínicas basado en motores de reglas clínicas para la clasificación automatizada de la anemia en los pacientes en hemodiálisis permitió una segmentación efectiva de los pacientes mediante la identificación de perfiles clínicos con necesidades diferenciadas de tratamiento. La integración de este tipo de sistemas en la práctica clínica podría mejorar la observancia de las guías, optimizar el uso del hierro y de los agentes estimulantes de la eritropoyesis, y facilitar la identificación de condiciones subyacentes como la resistencia al tratamiento asociada al hiperparatiroidismo secundario grave. Los futuros estudios deberán evaluar el impacto del sistema de apoyo a las decisiones clínicas en términos de los resultados clínicos, el uso de recursos y la experiencia del equipo tratante.

La automatización de la clasificación de la anemia mediante motores de reglas clínicas, como el desarrollado en este estudio, facilita la estandarización de los criterios diagnósticos y, también, representa una oportunidad para optimizar la eficiencia de los flujos de trabajo clínico. Al automatizar la interpretación de parámetros hematológicos y de metabolismo mineral, se reduce la necesidad de la interpretación manual, se minimiza la variabilidad interprofesional y se fortalecen los procesos de toma de decisiones basadas en la evidencia.

Si bien en este estudio no se evaluó formalmente el impacto económico, la literatura sugiere que los sistemas de apoyo a las decisiones clínicas tienen el potencial de disminuir los costos asociados con el uso inadecuado de agentes estimulantes de la eritropoyesis, el hierro intravenoso y las hospitalizaciones evitables ^25^. Además, su integración en los registros médicos electrónicos facilita una mayor observancia de los protocolos estandarizados y contribuye a la seguridad del paciente ^1^^,^^10^.

Samal *et al.* adelantaron un ensayo clínico aleatorizado multicéntrico (15 clínicas, 184 médicos de atención primaria, ~2.000 pacientes) para manejar la hipertensión no controlada en pacientes con enfermedad renal crónica mediante un sistema de apoyo a las decisiones clínicas integrado en la historia clínica, que fue diseñado con principios de economía del comportamiento y un enfoque centrado en el usuario.

Dicho sistema brindaba recomendaciones personalizadas basadas en la evidencia (por ejemplo, iniciar o titular los inhibidores de la enzima convertidora de angiotensina o los antagonistas del receptor de angiotensina II) durante la consulta, mientras que el grupo control recibió la atención habitual (el sistema de apoyo se encontraba en "modo silencioso"). A los seis meses, los pacientes de los médicos que seguían las recomendaciones del sistema de apoyo tuvieron una reducción adicional de la presión sistólica, en comparación con el grupo control (-14,6 mm Hg *versus* -11,7 mm Hg; p = 0,005).

En conjunto, los hallazgos sugieren que la implementación de este sistema puede ayudar a una mejor gestión de la hipertensión no controlada en la enfermedad renal crónica y, potencialmente, a mejores resultados clínicos a nivel poblacional ^26^. Estos resultados revelan una problemática estructural frecuente en la práctica clínica: la existencia de guías clínicas por sí sola no garantiza su adecuada implementación: factores como la sobrecarga asistencial, la complejidad de los algoritmos, la inercia terapéutica y la variabilidad en la experiencia clínica, pueden contribuir a un cumplimiento limitado de las recomendaciones. En este contexto, el sistema de apoyo a las decisiones clínicas actúa como un puente entre la evidencia y la acción médica concreta, al presentar recomendaciones contextuales, específicas para cada paciente y en el momento oportuno de la atención.

En otro estudio, se evaluó una intervención multimodal (recordatorios en el punto de atención primaria mediante el sistema de apoyo a las decisiones clínicas y la facilitación de prácticas) para mejorar el cuidado de la enfermedad renal crónica en estadio 3 o 4 (30 clínicas, ~6.700 pacientes). Este enfoque -basado en el modelo TRANSLATE de atención crónica-logró ralentizar la progresión de la enfermedad renal crónica, lo que se evidenció en una disminución más lenta de la tasa de filtración glomerular estimada en el grupo con el sistema de apoyo, en comparación con el grupo control. También, se observó una mejoría en la tendencia de la hemoglobina glucosilada en los pacientes con diabetes. Los autores señalan que el éxito se atribuye a la combinación del sistema de apoyo con otras estrategias ^27^. En una revisión narrativa enfocada en los sistemas de apoyo a las decisiones clínicas para enfermedades crónicas no transmisibles, de 49 publicaciones analizadas, la mayoría reportaban sistemas de apoyo integrados al expediente clínico electrónico (65 %) y una aplicación destacada en el ámbito cardio-renal-metabólico. En general, este tipo de sistemas de apoyo mostraron un impacto positivo, con mejoras en los indicadores de control de calidad (69 %) y en los reportes de beneficios clínicos (41 %), al compararlos con la atención habitual. Entre las características más frecuentes de los sistemas de apoyo exitosos, estuvieron: guías de tratamiento automatizadas, alertas o señales *flagging* de las condiciones que requieren atención, herramientas de estimación de riesgo, apoyo diagnóstico, módulos educativos y facilidades para exportar datos.

La revisión concluye que tanto los sistemas de apoyo a las decisiones clínicas incorporados en las historias clínicas electrónicas como los que funcionan de manera independiente, han mostrado resultados en su mayoría positivos, lo que ha mejorado la atención por parte del personal de salud y presentado un alto potencial de adopción. Sin embargo, dado que los estudios son heterogéneos en diseño y contexto, se destaca la necesidad de más investigación longitudinal que aclare qué factores influyen en la adopción exitosa de estos sistemas de apoyo en entornos reales ^28^.

En concordancia con estas recomendaciones y reconociendo las brechas identificadas en la literatura, este estudio cumplió con las principales directrices metodológicas para el desarrollo de un sistema de apoyo a las decisiones clínicas basado en el conocimiento. Se utilizaron datos disponibles en el *Sistema de información de laboratorio* como única fuente estructurada, reconociendo su limitación frente a otras fuentes clínicas. Las reglas de decisión aplicadas fueron definidas y validadas por expertos, y estaban en concordancia con los protocolos institucionales y las prácticas locales de atención. El sistema se integró al flujo de trabajo del laboratorio sin aumentar la carga al usuario, ya que solo generaba alertas simples que eran enviadas por correo electrónico a los expertos clínicos (directores médicos de las unidades renales). Estas reglas fueron sometidas a validación retrospectiva con datos reales y, luego, se documentó el proceso completo de diseño, revisión y ajustes. Aunque no se llevó a cabo un ensayo clínico aleatorizado, este estudio representa una fase preliminar que permitirá diseñar una intervención futura más sólida, con indicadores clínicos y operacionales como medidas de evaluación.

La estandarización del diagnóstico de la anemia de pacientes en hemodiálisis mediante los sistemas de apoyo a las decisiones clínicas basadas en el conocimiento podría representar un paso concreto hacia la transformación digital en nefrología. Su integración progresiva tiene el potencial de mejorar la calidad de la atención, optimizar el uso de los recursos terapéuticos, y favorecer una medicina más personalizada y segura para los pacientes con enfermedad renal crónica.

La construcción e implementación de un sistema de apoyo a las decisiones clínicas basado en un motor de reglas clínicas explícitas, permitió la clasificación automatizada de los pacientes con anemia en hemodiálisis. Esta herramienta facilitó la identificación de perfiles clínicos diferenciados y de potenciales condiciones comórbidas como el hiperparatiroidismo secundario grave. Este sistema, fundamentado en guías de práctica clínica y alimentado con datos reales de laboratorio, demostró su capacidad para segmentar la población de forma estandarizada, objetiva y clínicamente relevante.

El uso del sistema de apoyo a las decisiones clínicas podría contribuir a mejorar la observancia de las recomendaciones internacionales para el manejo de la anemia, optimizar el uso de hierro intravenoso y de los agentes estimulantes de la eritropoyesis, y reducir la variabilidad en la toma de decisiones médicas. Además, al permitir la detección automatizada de combinaciones de riesgo clínico -como la coexistencia de déficit funcional de hierro e hiperparatiroidismo secundario grave-, el sistema podría usarse para generar intervenciones terapéuticas más personalizadas y oportunas.

Este estudio aporta evidencia sobre la factibilidad técnica y el valor clínico de integrar herramientas de apoyo, basadas en el conocimiento, en el contexto asistencial real. Los futuros estudios deberán evaluar el impacto del sistema de apoyo a las decisiones clínicas sobre los desenlaces clínicos, el uso de recursos y el comportamiento de los profesionales de la salud frente a las alertas generadas.
